# Potential for flexible lactate shuttling between astrocytes and neurons to mitigate against diving-induced hypoxia

**DOI:** 10.3389/fnana.2025.1607396

**Published:** 2025-06-13

**Authors:** Chiara Ciccone, Sari Elena Dötterer, Sigrid Vold Jensen, Cornelia Geßner, Alexander C. West, Shona H. Wood, David G. Hazlerigg, Lars P. Folkow

**Affiliations:** ^1^Arctic Chronobiology and Physiology Research Group, Department of Arctic and Marine Biology, UiT-The Arctic University of Norway, Tromsø, Norway; ^2^Institute of Forest Genetics, Johann Heinrich von Thünen Institute, Großhansdorf, Germany

**Keywords:** diving mammals, hooded seal, mitochondrial respiration, hypoxia, neuron, astrocyte, lactate shuttling

## Abstract

For most non-diving mammals, lack of O_2_ (hypoxia) has detrimental effects on brain function. Seals, however, display a series of systemic, cellular, and molecular adaptations that enable them to tolerate repeated episodes of severe hypoxia. One as yet unresolved question is whether seal neurons in part employ anaerobic metabolism during diving: the “reverse astrocyte-neuron lactate shuttle” (rANLS) hypothesis postulates that seal neurons, by shuttling lactate to the astrocytes, may be relieved (1) from the lactate burden and (2) from subsequent ROS-production as lactate is oxidized by astrocytes upon re-oxygenation after the dive. Here, we have investigated this possibility, through histological and functional comparisons of the metabolic characteristics of neocortical neurons and astrocytes from the deep-diving hooded seal (*Cystophora cristata*), using mice (*Mus musculus*) as a non-diving control. We found that seal astrocytes have higher mitochondrial density and larger mitochondria than seal neurons, and that seal neurons have an atypical and significantly higher representation of the monocarboxylate lactate exporter MCT4 compared to mouse neurons. Also, measurements of mitochondrial O_2_ consumption suggest that the aerobic capacity of primary seal astrocytes is at least equal to that of primary seal neurons. Transcriptomics data from seals vs. mice suggest that specific adaptations to the electron transport system in seals may contribute to enhance hypoxia tolerance. These observations are consistent with the rANLS hypothesis.

## 1 Introduction

The brain is a highly aerobic organ and accounts for up to 20% of the total resting oxygen (O_2_) consumption in mammals (Erecińska and Silver, 2001). The high dependence on O_2_ makes the brain extremely sensitive to hypoxia (O_2_ deficiency). Normally, ATP is produced by mitochondria through oxidative phosphorylation (OXPHOS) by the Electron Transport System (ETS). The ETS is located on the inner mitochondrial membrane (IMM) and is composed of four enzymatic complexes (I to IV). Complexes I, III, and IV create an electrochemical gradient of protons across the IMM, which is utilized by ATP synthase (also referred to as complex V) to generate ATP (Chance and Williams, 1956). In the brain, ATP is mainly used to maintain membrane potentials and support synaptic transmission, both being fundamental for neuronal signal transmission (Harris et al., 2012). The crucial role of O_2_ is to act as final electron acceptor at complex IV, and under hypoxic conditions the electron flow through the ETS is therefore compromised and OXPHOS is diminished. In this situation, ATP production through anaerobic glycolysis attains increasing importance, but over time it is wasteful, non-sustainable, and does not provide ATP in sufficient amounts to maintain normal cerebral metabolic needs, especially in highly aerobic cells like neurons. Thus, severe hypoxia will typically result in a state of ATP deficiency and dysfunction - and, eventually, cell death (Boutilier, 2001; Lutz et al., 2003).

Mammals that face hypoxia regularly, like diving species, have evolved numerous adaptations to cope with prolonged periods of O_2_ shortage. These include enhanced O_2_ storing and carrying capacity, O_2_ economy via cardiovascular adjustments (bradycardia and peripheral vasoconstriction) and hypometabolism (summarized in Blix, 2018). Despite these adaptations, deep-diving pinnipeds do experience severe hypoxemia on a regular basis, with arterial partial oxygen pressure (PaO_2_) dropping below 15-20 mmHg in both Weddell seals (*Leptonychotes weddellii*) (Qvist et al., 1986) and northern elephant seals (*Mirounga angustirostris*) (Meir et al., 2009). These values are well below the critical PaO_2_ of 25-30 mmHg, known to negatively affect brain function in non-diving mammals (Erecińska and Silver, 2001). The hooded seal (*Cystophora cristata*) is a deep-diving pinniped, too, that may stay submerged for almost 1.5 h, and has been recorded to dive as deep as 1,600 m (Andersen et al., 2013; Vacquie-Garcia et al., 2017), although typically diving to 100-600 m depth for durations of 5-25 min (Folkow and Blix, 1999). Hooded seals evidently possess intrinsic cerebral hypoxia defense mechanisms, since—unlike mouse *(Mus musculus)* neurons—their neurons preserve both membrane potential and the ability to generate action potentials *in vitro* even after >1 h in severe hypoxia (Folkow et al., 2008; Geiseler et al., 2016). One of several factors that appear to contribute to this remarkable cerebral hypoxia tolerance (summarized in Larson et al., 2014), is an unusual organization of the metabolic roles between seal neurons and astrocytes (Mitz et al., 2009).

In the non-diving brain, neurons and astrocytes have different and complementary metabolic roles: according to the “astrocytes-neuron lactate shuttle” (ANLS) hypothesis, astrocytes produce ATP mainly through glycolysis, therein converting pyruvate into lactate, which is shuttled to the neurons to fuel OXPHOS (Pellerin and Magistretti, 1994). The hypothesis implies that while neurons largely operate aerobically, astrocytes perform mainly anaerobic metabolism (Pellerin and Magistretti, 1994), which is reflected in a higher aerobic capacity in neurons than in astrocytes (e.g., Almeida and Medina, 1997). In hooded seals, however, immunohistochemistry studies show that two typical markers for oxidative metabolism, neuroglobin and cytochrome c (Wong-Riley, 1989; Bentmann et al., 2005), are both more highly expressed in astrocytes than in neurons (Mitz et al., 2009). In addition, seal brain astrocytes show high expression of LDHB (Hoff et al., 2016), the lactate dehydrogenase (LDH) isoenzyme that primarily converts lactate to pyruvate. These observations formed the basis for the “reverse ANLS” (rANLS) hypothesis, according to which seal neurons may partially employ anaerobic metabolism when experiencing severe diving-induced hypoxia, and then shuttle the produced lactate to the astrocytes, which in turn can effectively oxidize lactate via aerobic metabolic pathways upon surfacing and re-oxygenation after the dive (Mitz et al., 2009). Later transcriptome data show elevated gene expression of the lactate exporter *Mct4* in hooded seal cortical tissue (Hoff et al., 2017)—and importantly also in isolated hooded seal neurons (Geßner et al., 2022) – which implies neuronal lactate production, thus further supporting the rANLS hypothesis. However, direct comparisons of MCT4 levels, mitochondrial densities and oxidative capacities in seal astrocytes vs. neurons, are still lacking.

Here, we present data showing that hooded seals have higher mitochondrial density with larger-sized mitochondria in astrocytes than in neurons, and that primary cultures of seal astrocytes show high aerobic metabolic capacity. We moreover show that neurons of hooded seals exhibit high levels of MCT4, a transporter protein that is otherwise widely expressed across species in astrocytes and other cell types with a glycolytic profile (Halestrap, 2012). Together, these observations are consistent with the rANLS hypothesis.

## 2 Materials and methods

### 2.1 Animals

Hooded seals (*Cystophora cristata)* were captured in their breeding colonies on the pack ice of the Greenland Sea, at ∼71°N and ∼019° W, during a research cruise with the R/V Helmer Hanssen in late March 2022 under permits of relevant Norwegian and Greenland authorities. Four adult lactating females were euthanized during the cruise, while six weaned pups were brought to the Department of Arctic and Marine Biology (AMB) at UiT—The Arctic University of Tromsø, Norway, where they were maintained for other research purposes in a certified research animal facility [approved by the Norwegian Food Safety Authority (NFSA, approval no. 089)]. They were eventually euthanized, in May and June 2023 (at age 12-14 mo.), in accordance with permits issued by the NFSA (permits no. 29013 and 29080). All seals were euthanized using the same procedure: seals were sedated by intramuscular injection of zolazepam/tiletamine (Zoletil Forte Vet., Virbac S.A., France; 1.5 – 2.0 mg per kg of body mass), then anesthetized using an endotracheal tube to ventilate lungs with 2-3% isoflurane (Forene, Abbott, Germany) in air and, when in full surgical anesthesia, they were euthanized by exsanguination via the carotid arteries. After decapitation, the brain was immediately removed, immersed in ice-cold artificial cerebrospinal fluid (aCSF, 128 mM NaCl, 24 mM NaHCO_3_, 0.5 mM NaH_2_PO_4_, 3 mM KCl, 1 mM MgCl_2_, 10 mM D-glucose, 20 mM sucrose, 3.5 mM CaCl_2_), and further subsampled, within 5-8 min of decapitation.

Three adult mice (*Mus musculus*, strain C57BL6) were euthanized by cervical dislocation in accordance with Norwegian and EU legislation (Landbruks- og matdepartementet, 2015; European Parliament, Council of the European Union, 2010), as part of another research project (permit 01/23, issued by Department of Comparative Medicine (AKM), UiT). Their brain was removed immediately after euthanasia and likewise placed in ice-cold aCSF before further subsampling (within 1 min of decapitation).

### 2.2 Immunohistochemistry

Histological analyses of mitochondria were performed using visual cortex samples from three adult hooded seals, three juvenile seals and three adult mice (as controls). Analyses of monocarboxylate transporter 4 (MCT4) were conducted with the same adult seal and mice samples.

Subsamples of visual cortex were collected immediately after euthanasia and placed in ice-cold aCSF in a petri dish lined with dental wax, where they were cut into cubes of 1 cm^3^ (seals) and 1 mm^3^ (mice). Adult seal tissue was fixed overnight in 4% paraformaldehyde (PFA) in Phosphate-Buffered Saline (PBS; 140 mM NaCl, 2.7 mM KCl, 8.1 mM Na_2_HPO_4_, 1.5 mM KH_2_PO_4_). Juvenile seal and mouse tissue was fixed overnight in 8% PFA in PHEM (60 mM PIPES (2,2′-(Piperazine-1,4-diyl)di(ethane-1-sulfonic acid)), 25 mM HEPES (4-(2-hydroxyethyl)-1-piperazineethanesulfonic acid), 10 mM EGTA (egtazic acid), and 4 mM MgSO_4_*7H_2_0). All samples were fixed overnight and then transferred to 0.4% PFA + 0.01% NaN_3_ in PBS for long-term storage at 4°C.

Before cryosectioning, the fixed tissue was left overnight in PBS with 30% sucrose + 0.01% NaN_3_ and then embedded in Tissue-Tek (Tissue-Tek^®^ O.C.T. Compound). Then, 12 μM (for MCT4 staining) and 20 μM (for mitochondrial staining) tissue sections were cut on a cryostat (CM3050, Leica, Germany) at −20°C. The sections were washed 3×1^–2^ min with PBS at room temperature (RT) and mounted on positively charged microscopy slides (Superfrost™ Plus Gold Microscope Slides, Epredia™), which were dried and then stored at 4°C in an airtight box overnight, or in an airtight container at −20°C, until immunolabeling.

#### 2.2.1 Immunolabeling of mitochondria

The visual cortex sections (20 μM) were post-fixed with 4% PFA in PBS for 20 min and washed with PBS 3 × 5 min. For antigen retrieval, the slides were incubated in citrate buffer (0.1 M Na_3_C_6_H_5_O_7_ *2H_2_O in PBS, pH = 6) at 80°C for 30 min. After cooling to RT, the slides were washed with PBS 2 × 5 min. To reduce autofluorescence, they were incubated with 3% H_2_O_2_ + 10% methanol (MeOH) in PBS for 10 min, then washed with PBS 3 × 5 min and incubated with blocking solution BS-T (0.8% cold water Fish Skin Serum (#G7765, Sigma-Aldrich) + 0.1% Bovine Serum Albumin (#A6588, ITW reagents) + 0.2% Trion-X (#T8787, Sigma) in PBS) for 30 min at RT. The tissue sections were then incubated with the following primary antibodies, diluted in BS-T at 4°C overnight: TOMM20 (Rabbit monoclonal, 1:500, Abcam, ab186734) for mitochondria, GFAP (Mouse monoclonal, 1:300 (seals), 1:600 (mice), Sigma-Aldrich, G6171) for astrocytes and MAP2 (Chicken polyclonal, 1:2,500, Abcam, ab 92434) for neurons. After washing with PBS 3 × 10 min, the tissue was incubated with secondary antibody diluted in BS-T for 90 min at RT: AF 555 (Goat anti-rabbit, 1:500 (seals), 1:1,000 (mice), Thermo Fisher, A-21428) for TOMM20, AF-647 (Goat anti-mouse, 1:200 (seals), 1:500 (mice), Thermo Fisher, A-21235) for GFAP, and CF-647 (Goat anti-chicken, 1:500 (seals), 1:1,000 (mice), Abcam, ab4600179) for MAP2. The tissue was then again washed with PBS 3 × 10 min and incubated with 4′,6-diamidino-2-phenylindole (DAPI) in PBS at a 1 μL/mL concentration, before new wash with PBS 2 × 5 min. To quench autofluorescence, the sections were incubated with TrueBlack^®^ Lipofuscin Autofluorescence Quencher (#23007, Biotium) diluted 1:20 in 70% ethanol: the microscopy slides were dipped in the quencher for 30 s and then put directly in a container with PBS for washing (3 × 10 min). Additional washes with distilled water (1 × 2 min) were done before mounting coverslips on top of the tissue sections using 2.5% DABCO anti-fade solution (90% glycerol + 2.5% 1,4-Diazobicyclo-(2,2,2) octane (DABCO) in H_2_O) and nail polish.

From each animal (*n* = 9), ten visual cortex slices were immunostained for mitochondria (TOMM20), of which five were stained for astrocytes (GFAP) and five for neurons (MAP2).

#### 2.2.2 Mitochondrial quantification

From each section, three 500 × 500 μM z-stacks were acquired with a VS120 Slide Scanner (Olympus, Japan) at 40× magnification, using the same optimized exposure setting across all images. Each z-stack consisted of 5 z-slices with 1 μm distance and was used to create a z-projection that collapsed the three-dimensional data into a 2D image. The images were analyzed using the software *FIJI* (version 1.54f, 2023). Macros were created to automatically run the image analysis steps. Cell areas were defined as regions of interest (ROI) by thresholding the z-projections of the GFAP/MAP2 channel and saving the outlines of the thresholded area, which automatically selected all regions of cellular staining within an image. Cell ROIs were laid over the z-projection of the TOMM20 channel to identify the mitochondria only within the cell regions (Supplementary Figure 1). Mitochondrial density was calculated based on the quantification of the number of pixels stained by TOMM20, following an image analysis method based on Song et al. (2008) and Shihan et al. (2021). Median filter subtraction (pixel radius = 4) was applied to the TOMM20 channel, which then was auto-thresholded (Otsu, 1975). The “watershed” function was applied to separate aggregated particles (Schindelin et al., 2012). Further, when using the “analyze particles” function, the number of mitochondria within each cell ROI was recorded and their size measured based on calibrated pixel size. All particles < 0.4 μm^2^ were excluded, as these were regarded as pixel noise.

In total, 270 images were made from the scanned tissue sections. All images from the 5th section on each microscopy slide were removed, as they all had notably lower staining intensities.

#### 2.2.3 Immunolabeling of MCT4

The slides with visual cortex tissue sections (12 μm) were maintained at RT for 5 min, post-fixed in 1% PFA in PBS for 10 min, then washed with PBS 3 × 5 min. Slides were then immersed in citrate buffer at 37°C for 20 min for antigen retrieval and cooled to RT. Slides were washed with PBS for 2 × 5 min, permeabilized by 0.1% Triton X in PBS for 10 min and washed again with PBS for 3 × 5 min. Non-specific binding sites were blocked with BSG blocking buffer with 2.2% glycine (Sigma-Aldrich, #G7126), 0.8% cold water Fish Skin Serum, and 0.1% bovine serum albumin (ITW reagents, #A6588) diluted in PBS with 0.05% Tween 20, for 60 min at RT. Sections were then incubated with primary antibodies, diluted in BSG with 0.05% Tween 20 overnight at 4°C: MCT4 antibody (Rabbit polyclonal, 1:500 (seal), 1:600 (mouse visual cortex), Proteintech, # 22787-1-AP) for MCT4, GFAP (Mouse monoclonal, 1:450 (seals), 1:900 (mice), Sigma-Aldrich, G6171) for astrocytes and MAP2 (Chicken polyclonal, 1:3,000, Abcam, ab 92434) for neurons. Next day, slides were washed with PBS 3 × 10 min before incubation with secondary antibodies in BSG with 0.05% Tween 20 for 90 min at RT (MCT4: Alexa Fluor 555, ani-rabbit, Thermo Fisher, A21428, 1:500 (seal) and 1:1,000 (mouse); MAP2: Alexa Fluor 647, goat anti-chicken, SIGMA Aldrich, ab4600179, 1:500 (seal) and 1:1,000 (mouse); GFAP: Alexa Fluor 647, goat anti-mouse, Thermo Fisher, A21235, 1:200 (seal) and 1:1,000 (mouse)). Slides were then rinsed again with PBS 3 × 10 min and counterstained with DAPI (Sigma-Aldrich, #10236276001, 0.2 μL/mL) in PBS 5-10 min before rinsing with PBS 2 × 5 min. Slides were immersed in TrueBlack^®^ Lipofuscin Autofluorescence Quencher (Biotium, #23007) diluted 20× in 70% ethanol for 1 min, to quench autofluorescence, washed PBS 3 × 10 min and rinsed in double-distilled water for 2 min. Slides were mounted with coverslips with 2.5% DABCO anti-fade solution (Sigma-Aldrich, #290734). The specificity of the MCT4 antibody was tested in mouse liver and *musculus gastrocnemius* (see Supplementary Figure 2).

For immunostaining of the visual cortex, 10 sections from each animal (*n* = 3 adult seals, *n* = 3 adult mice) were stained for MCT4; five were co-stained for astrocytes using GFAP, and five for neurons using MAP2. Negative control sections were prepared by omitting the primary antibodies to verify the absence of non-specific staining by secondary antibodies. All sections were stained simultaneously following an identical protocol.

#### 2.2.4 Quantification of MCT4

Tissue sections were imaged using a VS120 Slide Scanner (Olympus, Japan) at 40× magnification, capturing z-stacks with 0.5⋅μm intervals over an area of at least 1 mm^2^. From each z-stack, a region containing at least 15 cells of the relevant type (neurons or astrocytes) were selected. A z-projection of four consecutive z-slices was then generated to collapse the data into a 2D image of these regions. All images were analyzed, and MCT4 representation quantified, using QuPath version 0.4.4 (Bankhead et al., 2017). In each image, cells were annotated manually based on the signal of the cell-specific marker, and then the signal MCT4 was used to assess fractional MCT4 area within the cell regions. The *fractional MCT4 area*, i.e., the area that showed staining above a predefined intensity threshold, was assumed to reflect the relative presence of MCT4 in the cell, based on methods previously used to assess relative expression of cellular markers (Dahlrot et al., 2018; Zhou et al., 2021; Markussen et al., 2024). To quantify the threshold intensity, the mean of three background intensity measurements within each image was multiplied by the signal-to-noise-ratio, following a method adapted from Markussen et al. (2024).

### 2.3 Transcriptome analysis

Using previously published datasets (SRA code: PRJNA785765; Geßner et al., 2022), we analyzed the expression of specific complex I and II-related genes (Table 1) in the transcriptomes of hooded seal and mouse neurons from the visual cortex. Gene expression is presented as Transcripts per Kilobase Million mapped reads (TPM) and *p*-values were corrected for multiple testing using the false discovery rate (FDR) (Benjamini and Hochberg, 1995). In this study, only genes with a TPM-value >0 for either the mouse or the seal were analyzed and were considered as differentially expressed if FDR <0.05.

**TABLE 1:** Expression of selected complex I associated genes in visual cortex neurons of hooded seal and mouse (data from Geßner et al., 2022).

	Fold change	FDR	Mouse TPM	Seal TPM	Gene name
MT-ND1	19.21	5.19 × 10^–67^	21.03	399.34	NADH:ubiquinone oxidoreductase core subunit 1
NDUFA9	9.98	1.88 × 10^–29^	0.46	4.60	NADH:ubiquinone oxidoreductase core subunit A9
NDUFS1	−2.59	3.24 × 10^–11^	38.09	14.64	NADH:ubiquinone oxidoreductase core subunit S1
NDUFA4L2	2.03	0.49	0.35	0.69	NADH:ubiquinone oxidoreductase core subunit A4L2
SDHA	1.96	0.0004	9.51	18.56	Succinate dehydrogenase subunit A
SDHB	−1.52	0.00018	178.70	117.18	Succinate dehydrogenase subunit B

FDR, False Discovery Rate; TPM, Transcripts Per Kilobase Million.

### 2.4 Primary astrocytic culture

Astrocytic cultures were established based on brain tissue from the 4 wild-captured adult female seals, following Levison and McCarthy (1992). Briefly, after removing the brain, a slice of visual cortex was placed in ice-cold aCSF (within 5-8 min of euthanasia) and cut with a scalpel blade into small pieces. The minced tissue suspension was incubated in a shaking water bath at 37°C for 10 min. After addition of 1 mL of 2.5% trypsin (Cat. No: 15090046. Thermo Fisher Scientific, Massachusetts, United States) the suspension was incubated for an additional 20 min at 37°C. Then, 10 mL of culture medium (Dulbecco’s Modified Eagle Medium (DMEM, D5796, Sigma) + 10% Fetal Bovine Serum (FBS, F7524, Sigma) + 1% penicillin-streptomycin [Pen-Strep, P4458, Sigma)] were added and the suspension was triturated 10-15 times with a 10-mL pipette. When the pellet had sedimented, the supernatant was passed through a 100 μm strainer (352360, Falcon) into a 50-mL Falcon tube. This was repeated 2 more times. The collected suspension was centrifuged at 100 g for 10 min. The supernatant was removed, and the pellet was resuspended in 10 mL of culture medium and passed through a 70 μm strainer (734-0003, Falcon), and transferred to a T75 flask adding 5 mL of fresh culture medium. Cells were incubated at 37°C/5%CO_2_. The culture medium was changed the day after isolation and every 3-4 days after that.

### 2.5 Primary neuronal culture

Primary neurons from the 6 captive juvenile hooded seals were isolated from samples of their visual cortex following Brewer and Torricelli (2007). Briefly, a slice of fresh visual cortex (sampled within 5-8 min after euthanasia) was placed in ice-cold aCSF and transferred to a cold stainless-steel matrix (69-2150-1, AgnThos.se), and 0.5 mm slices were cut with a sterile blade. The slices were moved to a 15-mL polyethylene terephthalate (PET) tube (430055, Corning) containing 10 mL of HABG buffer at 4°C [Hibernate- A (A1247501, Thermo Fisher) + 2% B27 (1754044, Thermo Fisher) + 0.25% Glutamax (35050-061, Gibco)] and was incubated for 8 min in a 30°C shaking water bath. Tissue was transferred to a 15-mL PET tube containing a papain solution [Hanks’ Balanced Salt Solution (HBSS, 88284, Thermo Fisher) + 1.1 mM EDTA + 0.5 mM Glutamax + 1 mg/mL papain (31190, Worthington Biochemical)] pre-warmed at 30°C. After 30 min incubation in the shaking water bath, the tissue was transferred to 4 mL HABG medium (pre-warmed at 30°C) in a 15-mL PET tube and let rest at RT under the hood for 5 min. Sterile fire-polished to 1 mm diameter siliconized glass pipettes (Cat.No. 612-1701, VWR, Germany) coated with Sigmacoat (SL2, Sigma) were used to triturate the tissue for about 45 s. After the tissue pieces settled, the supernatant was transferred to an empty 15-mL PET tube. The sediment was resuspended in 2 mL of HABG, and trituration was repeated until 12 mL of suspension were collected. Two density gradients were prepared with OptiPrep (D1556, Sigma), as described elsewhere (Brewer and Torricelli, 2007). Six mL of cell suspension were carefully layered on each gradient and then centrifuged at 800 g for 15 min. Only fraction 2 and 3 of the resulting gradient were collected and mixed with 10 mL of HABG in a 15-mL PET tube. The suspension was centrifuged twice (after resuspension of pellets) for 2 min at 200 g. The second pellet was resuspended in 8 mL NeurobasalA/B27 medium [Neurobasal A (10888022, Thermo Fisher) + 2% B27 + 0.5 mM Glutamax + 10 μG/mL gentamycin (15710064, Gibco) + 5 ng/mL human fibroblast growth factor 2 (FGF2; PHG0369, Thermo Fisher)], pre-warmed to 22°C. Cell viability was determined by diluting 1:1 20 μL of the suspension with Trypan blue solution (T8154, Sigma). Thereafter, 150 μL of cell suspension was placed on each pre-coated poly-D-lysine (100 μg/mL, P6407, Sigma) coverslip and incubated at 37°C/5%CO_2_ for 1 h. Liquid was removed and the coverslips were washed with 1 mL of HABG at 37°C twice. Each coverslip was then moved with sterile forceps to a well of a 24-well plate containing 400 μL of NeurobasalA/B27 pre-incubated at 37°C/5%CO_2_. Half of the medium was changed 4 days after plating and then every 7 days. At 14 days *in vitro*, cells were treated with 15 μM of cytosine β-D-arabinofuranoside (AraC, C6645, Sigma) to stop growth of glia cells.

### 2.6 Immunocytochemistry for identification of cell types

Cells were rinsed with PBS (D8537, Sigma) 3 × 1 min and fixed with 4% PFA for 20 min at RT on a shaker. After fixation, cells were washed 3 × 5 min with PBS and incubated for 1 h with PBS-T [PBS + 0.1% Tween20 (P1379, Sigma)] and for 2 h with blocking solution (PBS-T + 10% goat serum [G9023, Sigma) + 1% Bovine Serum Albumin (BSA, A2153, Sigma)]. Cells were rinsed 3 × 5 min with PBS-T and incubated overnight at 4°C with primary antibody mouse anti-GFAP (1:300, G6171, Sigma) for astrocytes, and chicken anti-MAP2 (1:1,000, ab32454, Abcam) for neurons, in PBS-T + 1% BSA + 1% goat serum. The subsequent day, cells were rinsed again with PBS-T (3 × 5 min) and incubated with secondary antibody goat anti-mouse AF555 (ab150114, Abcam, 1:1,000) and goat anti-chicken AF647 (SAB4600179, Sigma, 1:1,000) in PBS-T + 1% BSA + 1% goat serum for 1 h at RT and protected from light. After 3 × 5 min washes in PBS-T, cells were incubated for 15 min with Hoechst (62249, Thermo Scientific) for nuclei staining. Coverslips were mounted with mounting medium (90% glycerol + 2.5% DABCO in H_2_O), slides were allowed to dry overnight and then imaged with a confocal microscope (Zeiss LSM 800, 20× objective).

For neuronal cultures, percentages of positive cells for both markers (GFAP and MAP2) were calculated using the positive cell selection function in QuPath-0.4.3 (Copyright© 2018-2022 QuPath developers, The University of Edinburgh). The percentage of positive cells was calculated to confirm enrichment for either astrocytes or neurons.

### 2.7 High resolution respirometry

High resolution respirometry (HRR) measurements were made with two O2k high resolution respirometers (Oroboros Instruments, Innsbruck, Austria) and data were real-time recorded using the Oroboros DatLab Software (Oroboros Instruments).

Astrocytic cultures were grown in T75 culture flasks: at 90% confluency, cells were washed twice with PBS and trypsinised with Trypsin + EDTA (T4049, Sigma) for 2 min. Cells were resuspended in culture medium and centrifuged at 200 g for 5 min, and the pellet was resuspended in 2 mL of MiR05 (0.5 mM EGTA, 3 mM MgCl_2_, 60 mM K-lactobionate, 20 mM taurine, 10 mM KH_2_PO_4_, 20 mM HEPES, 110 mM sucrose, 1 g/L BSA; pH = 7). Cells were counted with an automated cell counter (TC20, BioRad) and added to the Oxygraph chambers to a final concentration of 0.5-0.6 × 10^6^ cells/mL in a final volume of 2.1 mL.

Neurons were prepared for HRR according to Antico et al. (2021), with few modifications. Cells were washed twice with PBS and trypsinised with Trypsin + EDTA for 8 min, resuspended in NeurobasalA and centrifuged at 300 g for 3 min. Cells were resuspended in 1 mL of MiR05, and cell number was determined with the automated cell counter. Cells were resuspended in the Oxygraph chambers to 0.1 ×10^6^ cells/mL in a volume of 2.1 mL.

The subsequent steps were followed for both cell types: Cell counting was performed a second time, after addition to the Oxygraph chambers, for data normalization. After determination of the cellular basal respiration (Ce), cells were permeabilized with digitonin (37008, Fluka, 4 μg/mL) and leak respiration at complex I (CI_L_) was assessed by adding pyruvate (P; P2256, Sigma, 5 mM) and malate (M; M1000, Sigma, 2 mM). Oxidative phosphorylation (OXPHOS) via complex I (CI_P_) was measured by adding ADP (117105, Calbiochem, 2.5 mM), followed by cytochrome c (c; C7752, Sigma, 10 μM) for membrane integrity evaluation. If, after cytochrome c injection, there was more than a 10% increase in respiration, samples were excluded from the analysis. Thereafter, succinate (S; S2378, Sigma, 10 mM) was added to measure OXPHOS via complex I and II (CI_P_ + CII_P_), followed by rotenone (Rot; R8875, Sigma, 0.5 μM) for inhibition of complex I and calculation of complex II OXPHOS (CII_P_), and carbonyl cyanide p-trifluoro-methoxyphenyl hydrazone (FCCP; C2920, Sigma, 0.5 μM) for uncoupled respiration. Optimal concentration of digitonin and FCCP were determined in separate experiments (Supplementary Figure 3), except for neurons, for which optimal FCCP was determined empirically during each experiment.

#### 2.7.1 Calculations of relative respiratory efficiency

Since different procedures were used to isolate seal astrocytes and neurons prior to HRR, their absolute respiration capacity cannot be compared directly. To allow comparison, we therefore computed their efficiency of mitochondrial respiration in relative terms.

In one approach, we calculated the relative contribution of complex I and complex II to total respiration, as major differences in the activity of these two complexes have been reported between cell types in the rat (*Rattus norvegicus domestica*) (Almeida and Medina, 1997; Kristián et al., 2006). The relative contribution of complex I was calculated as *CI*_*P*_/(*CI*_*P*_ + *CII*_*P*_), and that of complex II as *CII*_*P*_/(*CI*_*P*_ + *CII*_*P*_).

Finally, we calculated the relative coupling efficiency of complex I as (1−(*CI*_*L*_/*CI*_*P*_)) and the relative changes in oxygen flow after complex I stimulation (calculated in relation to Ce; i.e., ((*CI*_*P*_−*Ce*)*100/*Ce*) and complex II stimulation (calculated in relation to CI_P_; i.e., (((*CI*_*P*_ + *CII*_*P*_)−*CI*_*P*_)*100/*CI*_*P*_).

### 2.8 Statistical analyses

To compare mitochondrial density across *cell type* (neurons and astrocytes) and *animal group* (adult seals, juvenile seals and mice), we used a generalized linear mixed model (GLMM) with Poisson distribution. To compare mitochondrial sizes across *cell type* and *animal group*, we used a linear mixed model (LMM) with Gamma distribution. *Section-ID* nested in *Animal-ID* was included as random term to correct for potential autocorrelation of images belonging to a given section of a given animal. Data are presented as means ± 95% confidence interval (CI).

To compare the fractional MCT4 areas between cell types, a Shapiro-Wilk test was first used to test for normality, and a Levene’s test to test for equal variance. Based on confirmation of both, a one-sided two-sample *t*-test was used, with *p*-values <0.05 considered statistically significant. Data are presented as mean ± standard error (s.e.m.)

High Resolution Respirometry data were analyzed using one-way ANOVA and post-comparison Tukey HSD test. Data are presented as mean ± s.e.m.

All analyses were done in RStudio (version 4.2.1 or version 4.3.1, 2023). The R-package *glmmTMB* (Brooks et al., 2017) was used for modeling and *emmeans* (Lenth et al., 2023) for pairwise *post-hoc* comparison tests.

## 3 Results

### 3.1 Mitochondrial density and size

**FIGURE 1:**
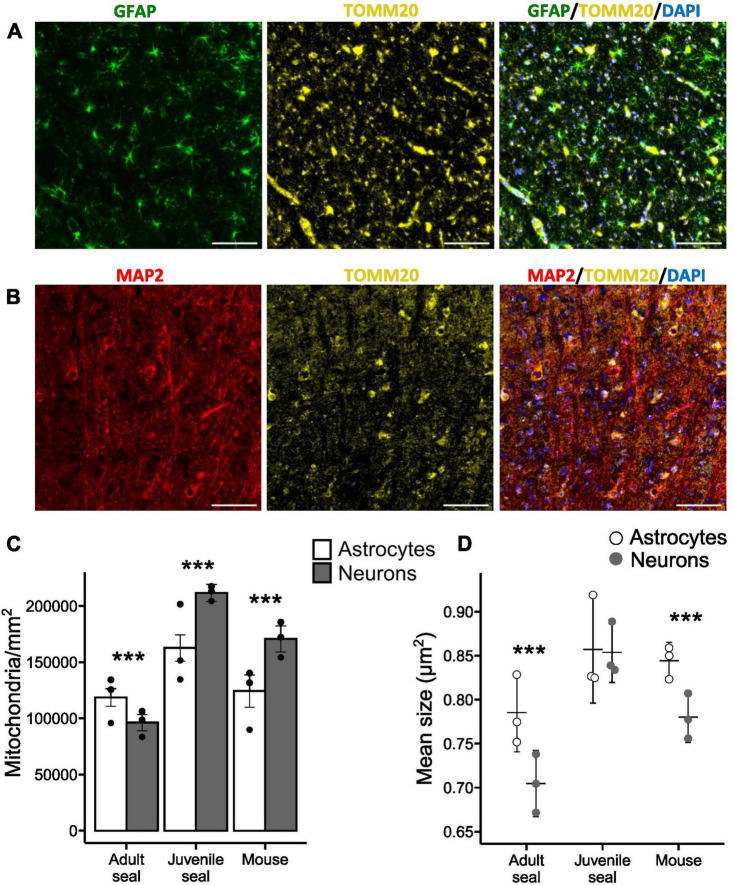
Mitochondria in neurons and astrocytes of the hooded seal visual cortex. **(A)** Adult hooded seal visual cortex imaged at 40×. Astrocytes in green (GFAP), mitochondria in yellow (TOMM20) and cell nuclei in blue (DAPI). **(B)** Adult hooded seal visual cortex imaged at 40×. Neurons in red (MAP2), mitochondria in yellow (TOMM20) and cell nuclei in blue (DAPI). **(C)** Counted mitochondria per mm^2^ of cell area. **(D)** Mean size of mitochondria (cross-sectional area). Scale bar = 100 μm. Bars/horizontal lines show the average per animal group and dots show the average of individuals. Error bars show the 95% confidence interval. ***Reflects statistically significant difference between cell types (*p* < 0.001).

Histological sections from the visual cortex were stained for astrocytes (GFAP), neurons (MAP2) and mitochondria (TOMM20) and used for mitochondrial analysis (Figures 1A,B). Adult hooded seals had significantly higher mitochondrial densities in astrocytes than in neurons (*p*< 0.001; Figure 1C), whereas the situation was reversed in juvenile seals (age ∼12-14 months) and mice (both *p*< 0.001; Figure 1C). Neuronal mitochondrial density was significantly higher in juvenile seals than in adults, with mouse neurons placed in between (all *p*< 0.001; Table 2). The mitochondrial density of astrocytes was also significantly higher in juvenile seals than in both adult seals and mice (both *p*< 0.001), while there was no difference between adult seal and mouse astrocytes (*p* = 0.987; Table 2).

**TABLE 2:** General linear mixed model (GLMM) of mitochondrial density.

Mitochondrial density							
	Estimate	SE		*Z*-value		*P*	
Animal group Adult seal (Intercept)	−2.13486	0.038834		−54.97		<0.001	
Animal group Juv seal	0.2656	0.054862		4.84		<0.001	
Animal group Mouse	0.035823	0.055358		0.65		0.518	
Cell type Neuro	−0.22104	0.005004		−44.17		<0.001	
Animal group Juv seal: Cell type Neuro	0.537009	0.006063		88.58		<0.001	
Animal group Mouse: Cell type Neuro	0.525206	0.010012		52.46		<0.001	
**Animal group**	**Cell type**	**lsmean**	**SE**	**df**	**asymp.LCL**	**asymp.UCL**	**Group**
Adult seal	Neuro	7.67	0.0388	Inf	7.59	7.74	a
Adult seal	Astro	7.89	0.0388	Inf	7.81	7.96	b
Mouse	Astro	7.92	0.0395	Inf	7.85	8	b
Juv seal	Astro	8.15	0.0388	Inf	8.08	8.23	c
Mouse	Neuro	8.23	0.0388	Inf	8.15	8.3	c
Juv seal	Neuro	8.47	0.0387	Inf	8.39	8.54	d
**Contrast**	**Estimate**	**SE**	**df**		**Z.ratio**		***P***
Adult seal astro—adult seal neuro	0.221	0.005	Inf		44.175		<.0001
Mouse astro—adult seal neuro	0.2569	0.05532	Inf		4.643		0.0001
Mouse astro—adult seal astro	0.0358	0.05536	Inf		0.647		0.9874
Juv seal astro—adult seal neuro	0.4866	0.05483	Inf		8.876		<.0001
Juv seal astro—adult seal astro	0.2656	0.05486	Inf		4.841		<.0001
Juv seal astro—mouse astro	0.2298	0.0553	Inf		4.155		0.0005
Mouse neuro—adult seal neuro	0.561	0.05483	Inf		10.232		<.0001
Mouse neuro—adult seal astro	0.34	0.05487	Inf		6.197		<.0001
Mouse neuro—mouse astro	0.3042	0.00867	Inf		35.069		<.0001
Mouse neuro—juv seal astro	0.0744	0.05481	Inf		1.357		0.7526
Juv seal neuro—adult seal neuro	0.8026	0.05478	Inf		14.651		<.0001
Juv seal neuro—adult seal astro	0.5816	0.05482	Inf		10.609		<.0001
Juv seal neuro—mouse astro	0.5457	0.05526	Inf		9.877		<.0001
Juv seal neuro—juv seal astro	0.316	0.00342	Inf		92.309		<.0001
Juv seal neuro—mouse neuro	0.2416	0.05476	Inf		4.411		0.0001

SE, Standard Error; df, degrees of freedom; lsmean, least squares mean; asymp.LCL, asymptotic lower confidence level; asymp.UCL, asymptotic upper confidence level.

The cross-sectional area of each counted mitochondria (adult seal total *n* = 176 375; juvenile seal total *n* = 417 503; mouse total *n* = 140 587) was used to calculate the mean mitochondrial size. Astrocytes of adult seals and mice had significantly larger mitochondria than had neurons (both *p*< 0.001; Figure 1D), while there was no difference in mitochondrial sizes between astrocytes and neurons of juvenile seals (*p* = 0.229; Figure 1D). The average size of mitochondria in adult seal astrocytes was 0.79 ± 0.042 (SD) μm^2^, which was significantly smaller than in astrocytes of juvenile seals (0.86 ± 0.05 μm^2^; *p*< 0.005) and mice (0.84 ± 0.06 μm^2^; *p*< 0.001), while there was no size difference in this respect between juvenile seals and mice (*p* = 1) (Figure 1D and Table 3). Average mitochondrial size for neurons was 0.70 ± 0.05 μm^2^, 0.85 ± 0.03 μm^2^ and 0.78 ± 0.06 μm^2^ in adult seal, juvenile seal, and mouse, respectively, being significantly different between all animal groups (all *p*< 0.001; Figure 1D and Table 3).

**TABLE 3:** Linear mixed model (LMM) of mitochondrial size.

Mitochondrial size							
	Estimate	SE		*Z*-value		*P*	
Animal group Adult seal (Intercept)	−0.235206	0.014573		−16.14		< 0.001	
Animal group Juv seal	0.077985	0.020578		3.79		< 0.001	
Animal group Mouse	0.081717	0.02084		3.92		< 0.001	
Cell type Neuro	−0.11697	0.002213		−52.85		< 0.001	
Animal group Juv seal: Cell type Neuro	0.120301	0.002674		44.98		< 0.001	
Animal group Mouse: Cell type Neuro	0.018564	0.004452		4.17		< 0.001	
**Animal group**	**Cell type**	**lsmean**	**SE**	**df**	**asymp.LCL**	**asymp.UCL**	**Group**
Adult seal	neuro	−0.352	0.0145	Inf	−0.381	−0.324	a
Mouse	neuro	−0.252	0.0145	Inf	−0.28	−0.223	b
Adult seal	astro	−0.235	0.0146	Inf	−0.264	−0.207	b
Juv seal	astro	−0.157	0.0145	Inf	−0.186	−0.129	c
Juv seal	neuro	−0.154	0.0145	Inf	−0.182	−0.125	c
Mouse	astro	−0.153	0.0149	Inf	−0.183	−0.124	c
**Contrast**	**Estimate**	**SE**	**df**		**Z.ratio**		***P***
Mouse neuro—adult seal neuro	0.10028	0.02056	Inf		4.877		<0.0001
Adult seal astro—adult seal neuro	0.11697	0.00221	Inf		52.853		<0.0001
Adult seal astro—mouse neuro	0.01669	0.02058	Inf		0.811		0.9657
Juv seal astro—adult seal neuro	0.19495	0.02056	Inf		9.482		<0.0001
Juv seal astro—mouse neuro	0.09467	0.02055	Inf		4.607		0.0001
Juv seal astro—adult seal astro	0.07798	0.02058	Inf		3.79		0.0021
Juv seal neuro—adult seal neuro	0.19829	0.02054	Inf		9.655		<0.0001
Juv seal neuro—mouse neuro	0.09801	0.02053	Inf		4.774		<0.0001
Juv seal neuro—adult seal astro	0.08132	0.02056	Inf		3.956		0.0011
Juv seal neuro—juv seal astro	0.00333	0.0015	Inf		2.219		0.2287
Mouse astro—adult seal neuro	0.19869	0.02082	Inf		9.543		<0.0001
Mouse astro—mouse neuro	0.09841	0.00386	Inf		25.478		<0.0001
Mouse astro—adult seal astro	0.08172	0.02084	Inf		3.921		0.0012
Mouse astro—juv seal astro	0.00373	0.02081	Inf		0.179		1
Mouse astro—juv seal neuro	0.0004	0.02079	Inf		0.019		1

SE, Standard Error; df, degrees of freedom; lsmean, least squares mean; asymp.LCL, asymptotic lower confidence level; asymp.UCL, asymptotic upper confidence level.

Mitochondrial size distribution analysis revealed a larger proportion of small mitochondria in adult seal neurons than in astrocytes, while there was no cell-specific size difference in either juvenile seals or mice (Supplementary Figure 4).

### 3.2 Fractional MCT4 area

**FIGURE 2:**
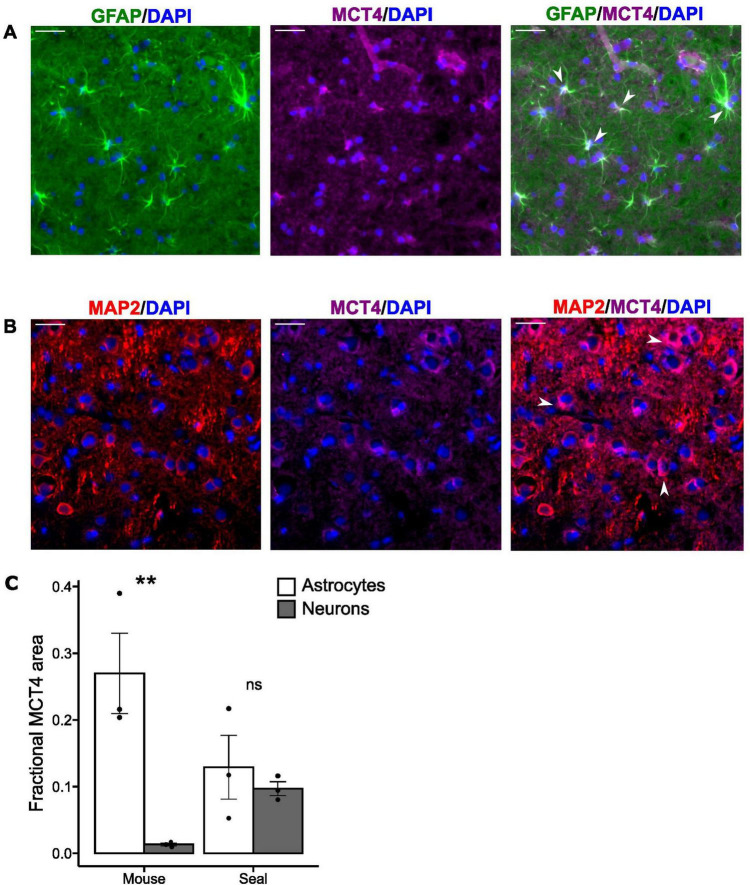
MCT4 is present both in astrocytes and neurons in the hooded seal brain. **(A)** Adult hooded seal visual cortex. Astrocytes in green (GFAP), MCT4 in magenta and cell nuclei in blue (DAPI). **(B)** Adult hooded seal visual cortex neurons in red (MAP2), MCT4 in magenta and cell nuclei in blue (DAPI). White arrows indicate specific cellular MCT4 signals within both astrocytes and neurons. **(C)** Mean fractional MCT4 area in astrocytes (white bars) and neurons (gray bars) from the visual cortex of mice and hooded seals. Data are presented as mean values ± s.e.m. Statistical significance is indicated with **(*p*<0.01). Scale bar = 30 μm.

A total of 225 cells from the visual cortex were analyzed for fractional MCT4 area within each cell type (astrocytes and neurons) and species [mice (*n* = 3) and hooded seals (*n* = 3)], with 75 cells being analyzed from each individual. In mice, fluorescence microscopy examination revealed specific MCT4 signal in astrocytes but not in neurons, while a specific MCT4 signal was present in both cell types in hooded seals (Figures 2A,B). Mouse astrocytes exhibited the highest fractional MCT4 area of all groups (0.270 ± 0.06), being significantly (*p* = 0.00649) >20-fold higher than in mouse neurons (0.013 ± 0.002) (Figure 2C). The average fractional MCT4 area in hooded seal astrocytes (0.129 ± 0.048) was not significantly higher (p<0.72) than in seal neurons (0.097 ± 0.001) (Figure 2C). The fractional MCT4 area in seal neurons was significantly higher (*p* = 0.0007), by >7-fold, than in mouse neurons. Mouse astrocytes fractional MCT4 area was higher than in seal astrocytes, although not statistically significant (*p* = 0.07).

### 3.3 Differential expression of complex I and II-associated genes in the hooded seal and mouse neurons from the visual cortex

**FIGURE 3:**
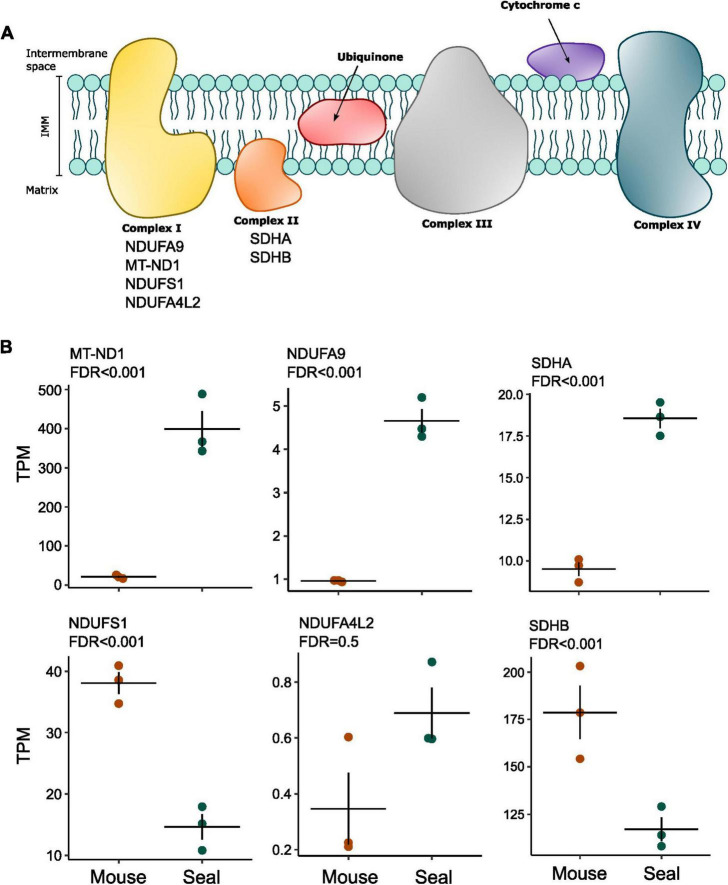
Differential expression of selected ETS genes between mouse neurons and seal neurons. **(A)** Schematic representation of the ETS complexes on the inner mitochondrial membrane (IMM). The list of selected genes for differential expression analysis is indicated under the respective complex name. **(B)** Expression levels are presented as transcripts per kilobase million (TPM). *P*-values were corrected for multiple testing using the false discovery rate (FDR) and presented as FDR in the figure. Horizontal lines show the average per animal group and dots show the average of individuals.

Given the involvement of different complex I subunits in the regulation of mitochondrial respiration (Tello et al., 2011; Babot et al., 2014; Lopez-Fabuel et al., 2016) and of complex II reverse activity in hypoxia and reoxygenation (Chouchani et al., 2014), we used an existing transcriptome dataset from laser captured hooded seal and mouse neurons (Geßner et al., 2022) to investigate the expression of genes associated with complex I and complex II (Table 1 and Figure 3A).

Two complex I subunits showed higher expression levels in seal neurons than in mouse neurons (Figure 3B): NADH:ubiquinone oxidoreductase core subunit 1 (MT-ND1; FDR = 5.19 × 10^–67^) (Geßner et al., 2022) and NADH:ubiquinone oxidoreductase core subunit A9 (NDUFA9; FDR = 1.88 × 10^–29^), both being associated with complex I deactivation (Babot et al., 2014). Although not significant (FDR = 0.49), NADH:ubiquinone oxidoreductase core subunit A4L2 (NDUFA4L2), a complex I subunit responsible for reduced complex I O_2_ consumption in hypoxia (Tello et al., 2011), also had higher expression values in seal neurons (Figure 3B). Moreover, the complex I NADH:ubiquinone oxidoreductase core subunit S1 (NDUFS1), associated with higher complex I-linked O_2_ consumption (Lopez-Fabuel et al., 2016), showed a reduced expression in the seal neurons (FDR = 3.24 × 10^–11^).

The complex II-linked subunit succinate dehydrogenase subunit A (SDHA) showed an increased expression in hooded seal neurons (FDR = 0.000392), while succinate dehydrogenase subunit B (SDHB), whose absence is connected to an increase in glutamate metabolism (Tabebi et al., 2022), was less expressed (Figure 3B) when compared to mouse neurons (FDR = 0.000183).

These results indicate significant differences between seal and mouse neurons in the gene expression of mitochondrial complexes I and II associated genes.

### 3.4 High resolution respirometry of primary astrocytic and neuronal cultures from hooded seal visual cortex

**FIGURE 4:**
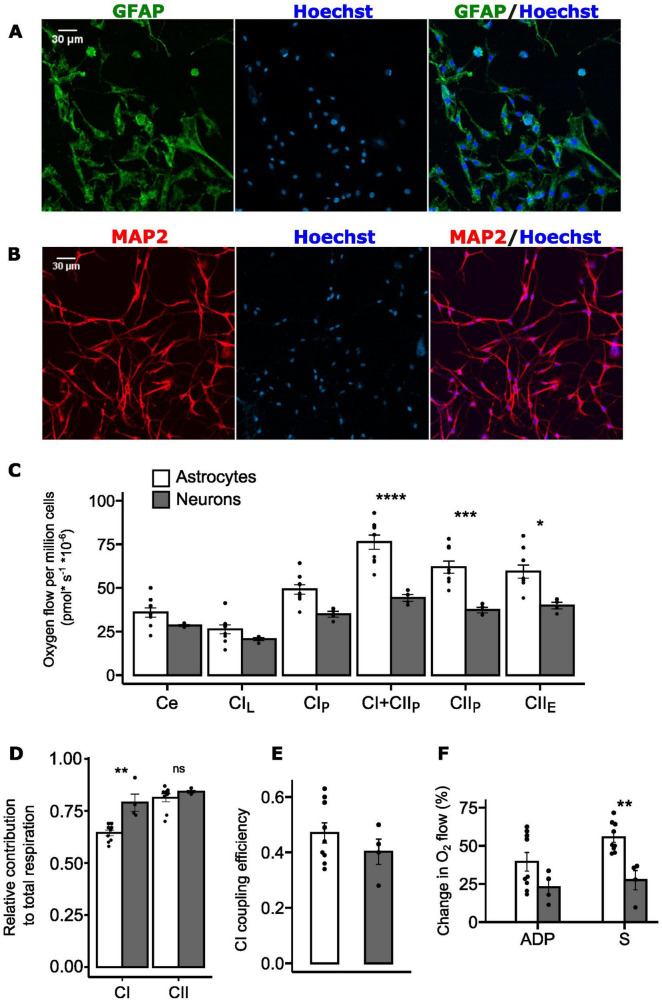
Complex I and complex II contribution to total mitochondrial respiration in primary astrocytes and neurons isolated from adult hooded seal visual cortex. **(A)** GFAP staining of primary astrocytes in green, Hoechst staining of nuclei in blue. Scale bar = 30 μm. **(B)** MAP2 staining of primary neurons in red, Hoechst staining of nuclei in blue. Scale bar = 30 μm. **(C)** Mitochondrial oxygen consumption of permeabilized primary astrocytes (white, *n* = 9) and neurons (gray, *n* = 4). Oxygen flow was normalized to cell number. Ce, basal cellular respiration; CI_L_, complex I leak; CI_P_, complex I OXPHOS; CI+CII_P_, complex I + complex II OXPHOS; CII_P_, complex II OXPHOS; CII_E_, complex II uncoupled state. **(D)** Relative contribution of complex I OXPHOS (CI_P_) and complex II OXPHOS (CII_P_) to total OXPHOS (CI+CII_P_). **(E)** Complex I (CI) coupling efficiency calculated as 1-(CI_L_/CI_P_). **(F)**. Relative change in O_2_ flow after ADP injection and after succinate (S) injection (*n* = 9 for astrocytes, *n* = 4 for neurons). Data are presented as mean ± s.e.m. ****p<0.0001, ***p<0.001, **p<0.01, *p<0.05 (one-way ANOVA).

The respiratory capacity of the mouse brain has been evaluated multiple times, clearly establishing that neurons have a higher oxidative capacity than astrocytes (Almeida and Medina, 1997; Kristián et al., 2006; Lopez-Fabuel et al., 2016). Our measurements of mitochondrial O_2_ consumption in hooded seal primary astrocytes (from wild-captured adult female hooded seals) and neurons (from captive juvenile, hooded seals; aged 12-14 months) provide the first corresponding data for a diving mammal. The isolated astrocytes, positive for GFAP staining (Figure 4A), showed the typical flat cell body and star-like morphology (Sun et al., 2017). Isolated neurons, positive for MAP2 staining (Figure 4B), had thinner shape, more rounded around the nucleus, with branching axons and dendrites. On average 92 ± 2.16% cells were MAP2 positive cells (Supplementary Figure 5A), indicating a neuronally enriched culture (Supplementary Figure 5B).

Overall, hooded seal primary astrocytes displayed higher respiratory rates than primary neurons (Figure 4C). However, since the cells were isolated from different age groups and were prepared and cultured under different conditions, they cannot be directly compared. Therefore, we focused on the relative contributions of complex I and II, since the activity of these complexes is significantly different between the two cell types in a non-diving mammal (Almeida and Medina, 1997).

When stimulated at the same time, complex I and complex II respiratory capacity tend to have an additive effect, thereby yielding elevated levels of total respiration (Lemieux et al., 2017). This may cause the relative contributions of complexes I and II to be apparently similar, when calculated separately (Lemieux et al., 2017). Nevertheless, these calculations can still give valuable insights on the complex-specific contribution. Here we show that neurons exhibit a higher *relative* contribution of complex I to total respiration (calculated as *CI*_*P*_/(*CI*_*P*_ + *CII*_*P*_)) compared to astrocytes (0.791 ± 0.042 and 0.645 ± 0.013, respectively; *p*<0.01; Figure 4D), while the relative contribution of complex II (calculated as *CII*_*P*_/(*CI*_*P*_ + *CII*_*P*_)) did not differ between the two cell types (0.813 ± 0.019 in astrocytes, 0.842 ± 0.006 in neurons; *p* = 0.343; Figure 4D).

We therefore calculated the complex I coupling efficiency 1-*L*/*P*-a high efficiency being indicative of high coupling of oxidation to the synthesis of ATP - but found no significant differences (*p* = 0.317) between seal astrocytes (0.47 ± 0.036) and neurons (0.40 ± 0.045) (Figure 4E).

Given these findings we compared the responses to stimulation of complex I (via ADP) and complex II (via succinate) and found the O_2_ flow after addition of ADP (i.e., CI_P_) to be higher in astrocytes (39.5 ± 6.09%) than in neurons (22.8 ± 5.03%), although not significantly so (*p* = 0.28; Figure 4F). However, the change in O_2_ flow upon the addition of succinate (i.e., CII_P_) was significantly higher in astrocytes (55.5 ± 3.26%) than in neurons (27.4 ± 6.27%; *p* < 0.01; Figure 4F).

## 4 Discussion

How the brain of deep-diving seals can endure extreme diving-induced hypoxemia (Qvist et al., 1986; Meir et al., 2009) has intrigued scientists for decades (e.g., Scholander, 1940; Elsner et al., 1970; Larson et al., 2014; Blix, 2018). In this context, research on the deep-diving hooded seal has shown that its remarkable cerebral tolerance to hypoxia is partly due to systemic adaptation [e.g., a redistribution of cardiac output that favors the brain, a brain cooling mechanism, enhanced cerebral capillary density (Scholander, 1940; Blix et al., 2010; Blix, 2018; Ciccone, 2019)], but also to intrinsic, cell-specific molecular and biochemical adaptations (e.g., Folkow et al., 2008). Immunohistochemical findings showing that two markers of high aerobic activity, neuroglobin and cytochrome c (Wong-Riley, 1989; Bentmann et al., 2005), unexpectedly were localized primarily in seal astrocytes and less in neurons led to the formulation of the “reverse ANLS hypothesis” (rANLS; Mitz et al., 2009), according to which seal neurons may cope with severe hypoxia by resorting to anaerobic metabolism, and avoid problems with lactate build-up by shuttling the lactate to the astrocytes. The astrocytes, with their higher aerobic capacity, would be well-equipped to metabolize the lactate upon surfacing and re-oxygenation, thereby also relieving neurons from oxidative stress (Mitz et al., 2009). The hypothesis gained support from observations by Hoff et al. (2016) showing that hooded seal astrocytes (unlike mouse astrocytes) show high expression of LDHB, which catalyzes conversion of lactate to pyruvate, and by Geßner et al. (2022) showing that isolated hooded seal neurons display elevated gene expression of *mct4*, a primarily lactate-exporting monocarboxylate transporter (Halestrap, 2012). In the present study, we provide additional histological and functional data on the metabolic roles of seal brain astrocytes and neurons that lend further support for the rANLS theory. Specifically, we have established primary cultures of hooded seal neurons and astrocytes and show that they present different mitochondrial O_2_ consumption profiles, which are also reflected in cell-specific mitochondrial densities and sizes. We have also used immunostaining to study the distribution and representation of the lactate-exporting monocarboxylate transporter MCT4 (Halestrap, 2012) in hooded seal neurons and astrocytes.

### 4.1 Mitochondrial density and cell-specific O_2_ consumption measurements suggest a high aerobic metabolic efficiency in adult hooded seal astrocytes

Highly aerobically active cells, like neurons and muscle cells, are generally rich in mitochondria, whereas mitochondria are found at lower densities in many other cells (Wong-Riley, 1989; Harris et al., 2012). Mouse astrocytes, which primarily rely on anaerobic metabolism typically have significantly *lower* mitochondrial densities than mouse neurons (Wong-Riley, 1989; Alano et al., 2007), which we confirm (Figure 1C). In contrast, astrocytes of adult hooded seals had significantly *higher* mitochondrial densities than had their neurons, i.e., the opposite to the expected mammalian mitochondrial distribution (Figure 1C), which is in accordance with immunolabeling data for cytochrome c in the seal brain (Mitz et al., 2009).

Mitochondria are organelles of high plasticity that can adapt morphologically and functionally to meet the metabolic needs of the cell (Westermann, 2012). Their size is positively correlated with cytochrome c oxidase activity, indicating that cells with larger mitochondria have a higher OXPHOS capacity than those with smaller-sized mitochondria (Wong-Riley, 1989; Westermann, 2012). We found the average mitochondrial size to be larger in astrocytes than in neurons, in both adult seals and mice (Figure 1D), indicative of higher rates of aerobic metabolism in astrocytes. Since mouse astrocytes primarily operate anaerobically (Walz and Mukerji, 1988; Bolaños et al., 1994), this weakens the argument that average mitochondrial size is a good proxy for aerobic metabolic capacity. However, aerobic capacity is linked to both numbers and size of mitochondria, and as stated, mouse astrocytes had significantly lower mitochondrial density than mouse neurons (Figure 1C).

Our high-resolution respirometry data show a higher - or at least similar - coupling efficiency, in astrocytic complex I compared to neuronal complex I (Figures 4C-F). This contrasts to murine neurons, which show significantly higher complex I coupling efficiency compared to astrocytes (Almeida and Medina, 1997; Kristián et al., 2006; Lopez-Fabuel et al., 2016). Our data therefore support that neurons and astrocytes of the seal brain have more flexible metabolic characteristics than have these cells in the typical non-diving brain.

Both the mitochondrial density data, and the respirometry results here reported are, thus, compatible with the rANLS hypothesis, which predicts that seal astrocytes have a high aerobic capacity (Mitz et al., 2009; Hoff et al., 2016). However, seal neurons must obviously maintain a highly functional oxidative system, since for most of the time they will have sufficient supply of oxygen. This is reflected in previous analyses showing increased expression of genes related to OXPHOS and ETS in seal neurons compared to mouse neurons (Geßner et al., 2020). It is also possible that, in order to maintain OXPHOS for as long as possible when O_2_ is scarce, seal neurons can lower their oxidative rate so as to make a more efficient use of the available O_2_ and thereby provide ATP for an extended period of time—possibly in combination with hypometabolism (e.g., Larson et al., 2014). Neuronal transcriptome data suggest that the synaptic activity of seal neurons is constitutionally reduced (Geßner et al., 2022). Indeed, synaptic signaling has been identified as the most energy-consuming processes of the brain (Harris et al., 2012), implying that its depression would reduce cellular ATP demands and, thereby, the need to maintain a high mitochondrial O_2_ consumption rate in neurons. Neural spontaneous activity, as well as hippocampal synaptic signaling, have been demonstrated to be depressed during hypoxia exposure in *in vitro* electrophysiological experiments with fresh hooded seal brain tissue (Ramirez et al., 2011; Czech-Damal et al., 2014; Geiseler et al., 2016). In the brain of other hypoxia-tolerant species [red-eared slider turtles (*Trachemys scripta elegans*) and naked-mole rats (*Heterocephalus glaber*)], exposure to anoxia/hypoxia reduces complexes I and II activity (Pamenter et al., 2016, 2018), further demonstrating the important role of depressing OXPHOS rate in order to convey hypoxia tolerance.

We found that the relative contribution to total respiration of complex I was higher for seal neurons than for seal astrocytes (Figure 4D), possibly indicating a greater relative capacity of complex I in neurons than in astrocytes. According to the rANLS hypothesis, astrocytes are suggested to have at least as high an aerobic capacity as have neurons, which is here supported by their significantly higher mitochondrial density compared to neurons in the adult hooded seal (Figure 1C), and their higher CI coupling efficiency (Figure 4E). In this perspective, and considering the involvement of complex I in ROS production upon reoxygenation (Murphy, 2009), the reduced dependency on complex I activity of seal astrocytes (Figure 4D) may represent an adaptation to diving-induced hypoxia, since both hypoxia and anoxia have been documented to reduce complex I activity, while still maintaining ETS integrity and functionality in mice (Spinelli et al., 2021; Ravasz et al., 2024).

### 4.2 A high fractional representation of MCT4 in seal neurons suggests that they may resort to anaerobic metabolism when needed

We have here shown that hooded seal visual cortex neurons contain more MCT4 than do mouse neurons, which hardly express this protein at all (Rafiki et al., 2003; Figure 2C). This is in line with data by Geßner et al. (2022), and also with the rANLS hypothesis (Mitz et al., 2009), implying that anaerobic seal neurons could rid themselves of lactate via MCT4. Interestingly, mouse neurons show elevated expression of MCT4 after ischemic episodes *in vivo* (Rosafio et al., 2016), and its expression appears to be induced by hypoxia, via activation of the HIF-1 pathway (Rosafio and Pellerin, 2014). The similar representation of MCT4 between seal neurons and astrocytes (Figure 2C) implies that conventional ANLS is employed also in the seal brain when sufficient amounts of O_2_ are available. Although transcriptome studies have been performed on brain tissue from other diving mammals (e.g., Krüger et al., 2020), these did not focus on the MCT4 distribution in the brain and, by using whole brain tissue, they could not have differentiated between neuronal vs. astrocyte MCT4 expression, anyway. Therefore, it is as yet unknown to what extent this trait is shared among different diving species.

### 4.3 Differential expression of complex I and II-associated genes in seals and mice may reflect different adaptations to hypoxia

Corroborating the hypothesis of a possibly reduced O_2_ consumption in seal neurons, we found a lower expression of the complex I subunit NDUFS1 in seal neurons than in mouse neurons (Table 1 and Figure 3B). In rats this subunit was found to be more abundant in neurons than in astrocytes, and its knock-out resulted in decreased complex I O_2_ consumption (Lopez-Fabuel et al., 2016). The low expression of NDUFS1 in seal neurons might then be causally linked to a reduced complex I O_2_ consumption of neurons (Figure 4E). Moreover, *in vitro* exposure of hooded seal visual cortex slices to a hypoxia/reoxygenation protocol was reported to cause further downregulation of NDUFS1 (Hoff et al., 2017), underlining its potential role in ETS regulation in seals.

Differences in mitochondrial O_2_ consumption in neurons and astrocytes of rats have been attributed to the presence of a higher ratio of deactive complex I in astrocytes compared to neurons. Instead of working as a NADH oxidoreductase, deactive complex I functions as a Na^+^/H^+^ antiporter mechanism (Roberts and Hirst, 2012). Many studies show that this functional switch is induced by hypoxia exposure (Galkin et al., 2009; Dröse et al., 2016; Stepanova et al., 2019), and it is thought to be beneficial for the maintenance of the ionic balance across the inner mitochondrial membrane (IMM). Deactive complex I presents some structural rearrangements which make three specific subunits more exposed than in the active form: NADH:ubiquinone oxidoreductase core subunit 3 (MT-ND3), MT-ND1 and NDUFSA9 (Babot et al., 2014). Although we found both MT-ND1 and NDUFSA9 to be more highly expressed in seal neurons than in mouse neurons (Figure 3B), it is difficult to conclude whether seal neurons have a higher proportion of deactive complex I, as we did not perform any structural or functional enzymatic assay. Considering that complex I deactivation seems to be one of the elements determining metabolic differences between astrocytes and neurons and its involvement in reducing ROS production upon reoxygenation (Lopez-Fabuel et al., 2016; Stepanova et al., 2019), it might be interesting to further investigate complex I characteristics in the pinniped brain in future studies.

Both the complex II subunits analyzed here (succinate dehydrogenase complex, subunit A (SDHA) and B (SDHB)) show differential expression in seal neurons compared to mouse neurons, with SDHA being more highly expressed and SDHB being less expressed in seal neurons (Figure 3B). Increased levels of SDHA were already reported in rats with high hypoxia tolerance after exposure to moderate hypoxia, together with increased succinate levels (Mironova et al., 2019). Succinate is indeed known to accumulate during hypoxia and to contribute to the cellular hypoxia response (Selak et al., 2005; Chouchani et al., 2014). Although we did not measure succinate levels, it is possible that SDHA might be contributing to ischemic succinate accumulation also in seal neurons, enhancing glycolytic metabolism. The low SDHB expression might also be influencing neuronal cellular metabolism, as its knock-out in cancer cells diverts metabolism toward glutamate catabolism and glycolysis (Cardaci et al., 2015; Tabebi et al., 2022).

### 4.4 Mitochondrial size and densities suggest age-related changes of aerobic metabolic capacity in hooded seal brain neurons

We observed age-related differences in mitochondrial densities and sizes in the hooded seal brain; with adult seal neurons having lower densities and smaller size than in juveniles (Tables 1, 2). We also found that juvenile seals have a similar cell-specific mitochondrial distribution to that of mice (Figure 1C), with significantly higher densities in neurons than in astrocytes (Figure 1C). This suggests that there is an age-related transition in dependence on aerobic metabolism in neurons and astrocytes in hooded seals, possibly indicating that neurons rely more on aerobic metabolism at young age, when the seals do not dive as deep and as long as they do as adults (Folkow et al., 2010). Such developmental changes have been demonstrated in other hypoxia-related adaptations in pinnipeds, e.g., myoglobin levels (Burns et al., 2007; Geiseler et al., 2013).

## 5 Conclusion and perspectives

Our data show cell-specific differences in brain mitochondrial function and organization, and in MCT4 distribution, between diving and non-diving mammals. “Diving neurons” appear to tolerate episodes of hypoxia by having a lower O_2_ consumption capacity and being able to resort to anaerobic glycolysis. Our mitochondria counts and respirometry data suggest a higher aerobic efficiency in adult hooded seal astrocytes than in neurons. These findings are all in accordance with the rANLS hypothesis (Mitz et al., 2009). Based on the evidence presented in this study, we further propose that the hypoxia tolerance of hooded seal neurons involves a reduced and more efficient use of O_2_, possibly via molecular rearrangements of mitochondrial complex I and II subunits and aided by the ability of astrocytes to remove, and later metabolize, lactate (Hoff et al., 2016). To shed more light on these mechanisms, future studies should attempt to perform hooded seal astrocyte-specific transcriptome analyses, to better understand the metabolic adaptations and roles of these cells and their possible contributions to cerebral hypoxia tolerance and antioxidant defense in seals. Also, to improve the understanding of how mitochondrial morphology relates to respiration, it is important to further investigate mitochondrial ultrastructure, especially the organization of the IMM, previously shown to be linked to OXPHOS rate (Kristián et al., 2006).

### 5.1 Limitations to the study

For logistic reasons, our respirometry data were collected from two different age groups of hooded seals; adults for the astrocytes and juveniles (∼1 year old) for neurons: the establishment of primary neuronal cultures requires a highly stable and sterile environment, which was not achievable on a research vessel in the Arctic Greenland Sea, which is why we could not conduct respirometry experiments with adult seal primary neurons.

## Data Availability

The original contributions presented in the study are publicly available. This data can be found here: https://doi.org/10.18710/FRCOQZ.
