# Supplementary material for: Potential for flexible lactate shuttling between astrocytes and neurons to mitigate against diving-induced hypoxia
Source: Front Neuroanat. 2025 Jun 13;19:1607396. doi: 10.3389/fnana.2025.1607396 (PMC12202495; doi:10.3389/fnana.2025.1607396)
Supplement: Supplementary file 1 [file Table_1.docx]

***Supplementary Material***

***
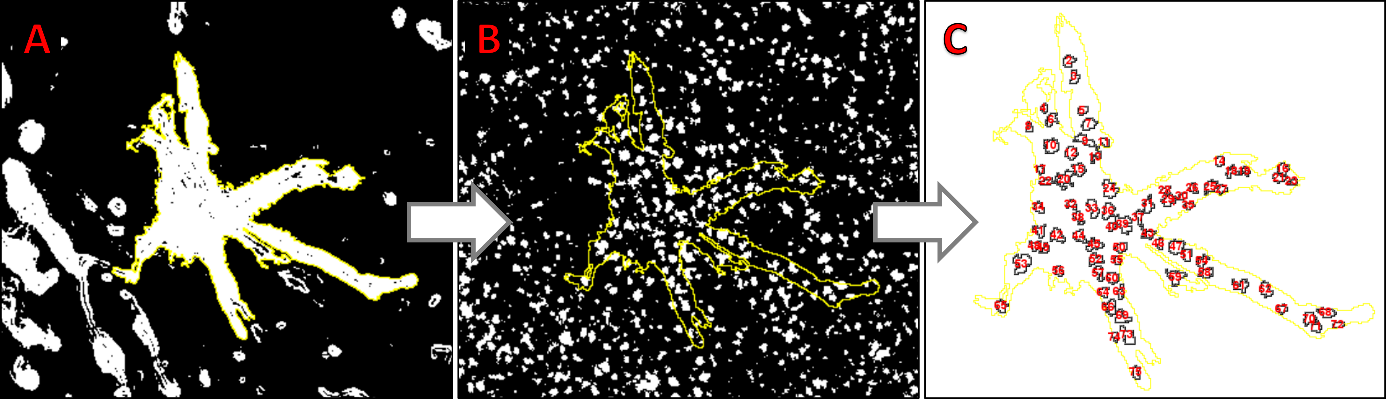
***

**Figure S1. Example of image analysis workflow in assessing mitochondrial densities. (A)** Region of interest (ROI); here, an astrocyte, created from the GFAP channel, **(B)** overlaying the median filter subtracted TOMM20 channel, **(C)** detecting the mitochondria numbered in red.

**Figure S2. Positive immunolabelling control for the MCT4 antibody.** Sections of mouse liver **(A)** and *musculus gastrocnemius* **(B)** stained for MCT4 (red) and cell nucleus with DAPI (blue). No specific signal is observed in the liver **(A)**, whereas a strong signal is present in gastrocnemius muscle fibres **(B)**. Scale bar: 40 μm. Method: Two adult mice (*Mus musculus*, strain C57BL6) were euthanized by intraperitoneal injection of an overdose of pentobarbital (100 mg/kg) followed by exsanguination, in accordance with Norwegian and EU legislation (Landbruks- og matdepartementet, 2015; European Parliament, Council of the European Union, 201 0), as part of other research projects (permit 18/21, issued at Department of Comparative Medicine (AKM), UiT). Their liver and musculus gastrocnemius were used as negative (Dimmer et al., 2000) and positive (Chen et al., 2023; Halestrap & Meredith, 2004) controls for MCT4. Immunohistochemistry and immunolabelling of MCT4 was performed as described in the main text, using mouse liver (cut in 1 mm3 cubes) and mouse gastrocnemius muscle (intact, to preserve muscle fibre organization), both fixed overnight in ice-cold fixative (4 % PFA in PBS) within 8 minutes after euthanasia, then transferred to 0.4 % PFA + 0.01 % NaN3 in PBS for long-term storage at 4°C before sectioning (20 μm thick sections) The specificity of our MCT4 antibody is confirmed from the positive immunolabeling (specific signal) of MCT4 in mouse *musculus gastrocnemius* (mean fluorescence intensity (MFI) = 878 ± 93 a.u.) and the absence of specific signal in mouse hepatocytes (MFI = 89 ± 23 a.u.) (Fig. S1).


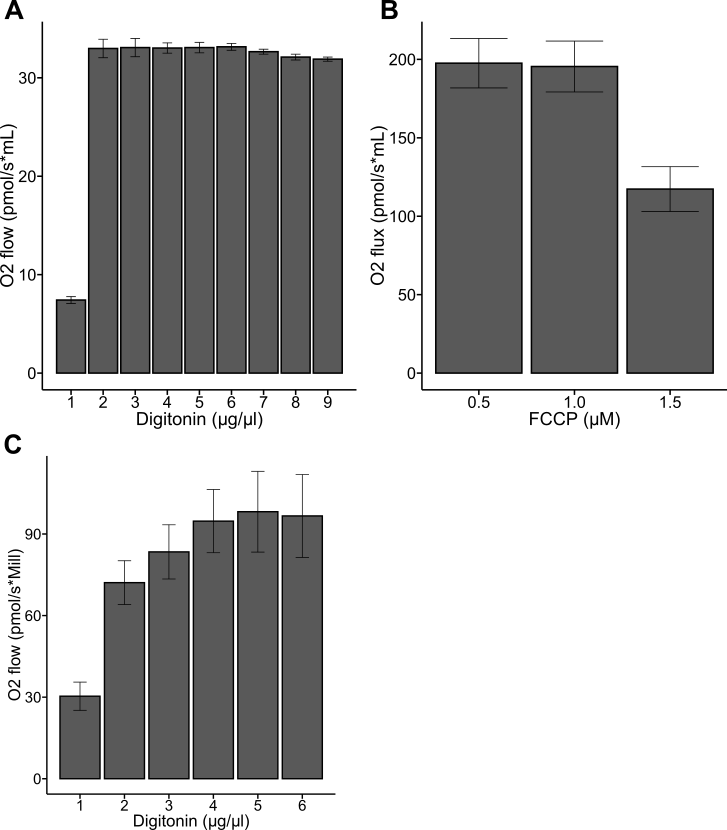


**Figure S3. Optimal concentration of digitonin and FCCP for astrocytes and neurons. A)** Optimal digitonin concentration was determined for astrocytes following Doerrier et al. (2018). After 9 µg/µl of digitonin, cells did not pass the cytochrome c test (respiration increased over 10%). A final concentration of 4 µg/µl was chosen as it was the lowest concentration at which all cells showed maximal respiration. Data are presented as mean ± s.e.m. (n=4) **B)** Optimal FCCP concentration for astrocytes. Severe reduction in respiration was seen already at 1.5 µM FCCP. At 1 µM FCCP, cells showed different responses: in some samples there was already a decrease in respiration, in others there was a small increase. To avoid possible inhibition of respiration in some of the samples, we used 0.5 µM as optimal FCCP concentration. Data are presented as mean ± s.e.m. (n=9, except n=2 for 1.5 µM of FCCP). **C)** Optimal digitonin concentration for seal neurons. At 6 µg/µl digitonin, cells did not pass the cytochrome c test. During a first experimental procedure, 5 µg/µl were used but the data had to be excluded since cells did not pass the cytochrome c test again. Decreasing the use of digitonin concentration to 4 µg/µl did not damage the cells and it was used as optimal concentration for the neurons. Data are presented as mean ±s.e.m (n=3).


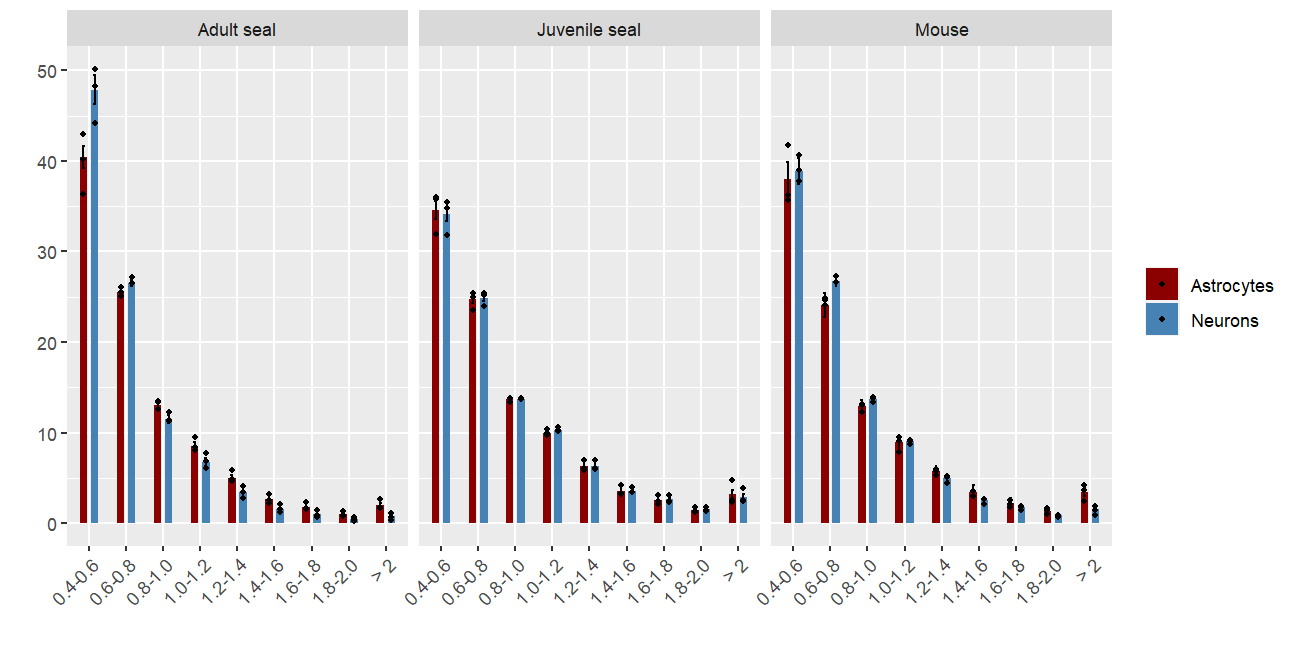


**Figure S4. Average size distribution of mitochondria.** % mitochondria = mitochondria within size range / (total number of mitochondria * 100). Bars show the average per animal group and black dots show the average of individuals. Error bars show the 95% confidence interval.


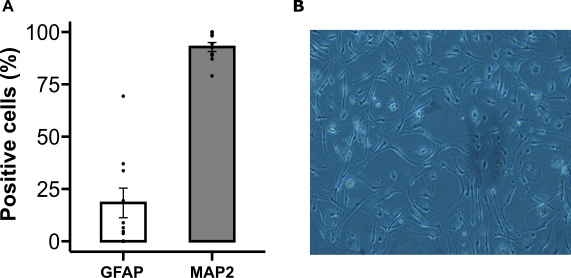


**Figure S5. Percentage of MAP2 positive cells in neuronal cultures. (A)** Percentage of GFAP or MAP2 positive cells in primary neuronal cultures (*n*=10) before treatment with AraC. Data are presented as mean ± s.e.m. **(B)** Inverted microscope image of a primary neuronal culture after 7 days incubation with AraC (15 µM) and 30 minutes before being processed for HRR.

**References:**

**Chen, Z., Bordieanu, B., Kesavan, R., Lesner, N. P., Venigalla, S. S. K., Shelton, S. D., DeBerardinis, R. J. and Mishra, P.** (2023). Lactate metabolism is essential in early onset mitochondrial myopathy. Sci. Adv., **9(1)**, eadd3216.

**Dimmer, K. S., Friedrich, B., Lang, F., Deitmer, J. W. and Bröer, S.** (2000). The low-affinity monocarboxylate transporter MCT4 is adapted to the export of lactate in highly glycolytic cells. *Biochem. J.*, **350 Pt 1(1)**, 219-227.

**Doerrier, C., Garcia-souza, L. F., Krumschnabel, G., Gnaiger, E., Wohlfarter, Y.** (2018). High-Resolution FluoRespirometry and OXPHOS Protocols for Human Cells, and Isolated Mitochondria. *Mitochondrial Bioenergetics: Methods and Protocols*. 1782(31-70)

**European Parliament, Council of the European Union (**2010) Directive 2010/63/EU of the European Parliament and of the Council of 22 September 2010 on the protection of animals used for scientific purposes http://data.europa.eu/eli/dir/2010/63/oj

**Halestrap, A. P. and Meredith, D.** (2004). The SLC16 gene family-from monocarboxylate transporters (MCTs) to aromatic amino acid transporters and beyond. Pflugers Arch, 447(5), 619-628.

**Landbruks- og matdepartementet** (2015) Forskrift om bruk av dyr i forsøk [in Norwegian; in English: Regulation of use of animals in research], (FOR-2015-06-18-761) https://lovdata.no/dokument/SF/forskrift/2015-06-18-761
